# Mechanisms and therapeutic perspectives of mitochondrial dysfunction of macrophages in periodontitis

**DOI:** 10.3389/fcimb.2025.1634909

**Published:** 2025-08-11

**Authors:** Yibing Jia, Zili Li, Pengjie Huang, Yan Wang, Bo Yang

**Affiliations:** Hospital of Stomatology, Guanghua School of Stomatology, Guangdong Provincial Key Laboratory of Stomatology, Sun Yat-sen University, Guangzhou, China

**Keywords:** mitochondrial dysfunction, macrophage polarization, osteoclast differentiation, periodontitis mechanism, periodontitis treatment

## Abstract

Periodontitis is a global inflammatory oral disease, and plaque-induced host excessive immune response is recognized as a major cause of its pathogenesis. In recent years, the relevance of mitochondrial dysfunction to periodontitis has been increasingly investigated, particularly with respect to macrophages, the key immune cells in the periodontal immune microenvironment. Mitochondrial dysfunction drives macrophage M1 polarization and osteoclast differentiation through mechanisms such as metabolic reprogramming, reactive oxygen species release, abnormal mitophagy, abnormal mitochondrial biogenesis and damaged mitochondrial dynamic. In addition, mitochondrial transfer in the periodontitis setting has been reported in several researches. In this review, we highlight the impact of mitochondrial dysfunction on macrophages in the periodontitis setting and summarize emerging therapeutic strategies for targeting mitochondria in periodontitis, including antioxidants, modulators of metabolic reprogramming, nanomaterials and photodynamic therapy.

## 1 Introduction

Periodontitis is the leading cause of tooth loss in adults, affecting approximately 90% of the global population (Luo et al., 2024; Zhou et al., 2024). It is characterized by irreversible inflammatory damage to periodontal supporting tissues, destruction of gingival connective tissue and collagen, and alveolar bone resorption, ultimately leading to tooth loss (Kinane et al., 2017). If untreated, severe periodontitis may lead to serious consequences such as massive tooth loss, masticatory dysfunction, aesthetic impairement, decreased self-esteem and even social inequality (Herrera et al., 2022). However, current clinical periodontal treatment strategies present limitations such as the possibility of mechanical injury (Huang et al., 2025) and the neglect of dysregulated immune-inflammatory responses central to disease progression (Lin et al., 2022), which make it difficult to achieve the desired results. Therefore, periodontitis remains an important public health problem and an economic burden (Yang et al., 2021).

The etiology of periodontitis is complex. The excessive inflammatory and immune response that subgingival plaque induces in the host is recognized as a major cause of the pathogenesis of periodontitis (Bascones et al., 2005). Destruction of periodontal tissues by the pathogenic bacteria, coupled with the release of host pro-inflammatory cytokines, matrix metalloproteinases, and reactive oxygen species (ROS) leads to degradation of collagen fibers, resorption of the alveolar bone and collapse of the periodontal ligament (Dahiya et al., 2013).

Traditional views have focused on the etiology and pathogenesis of periodontal disease in specific bacterial species (Teles et al., 2022). However, with further investigation into the pathogenesis of periodontitis, it is currently considered that the interactions between the microbiome and its components cannot be ignored. In addition, interactions between different microbiomes have been elucidated.

**Figure 1:**
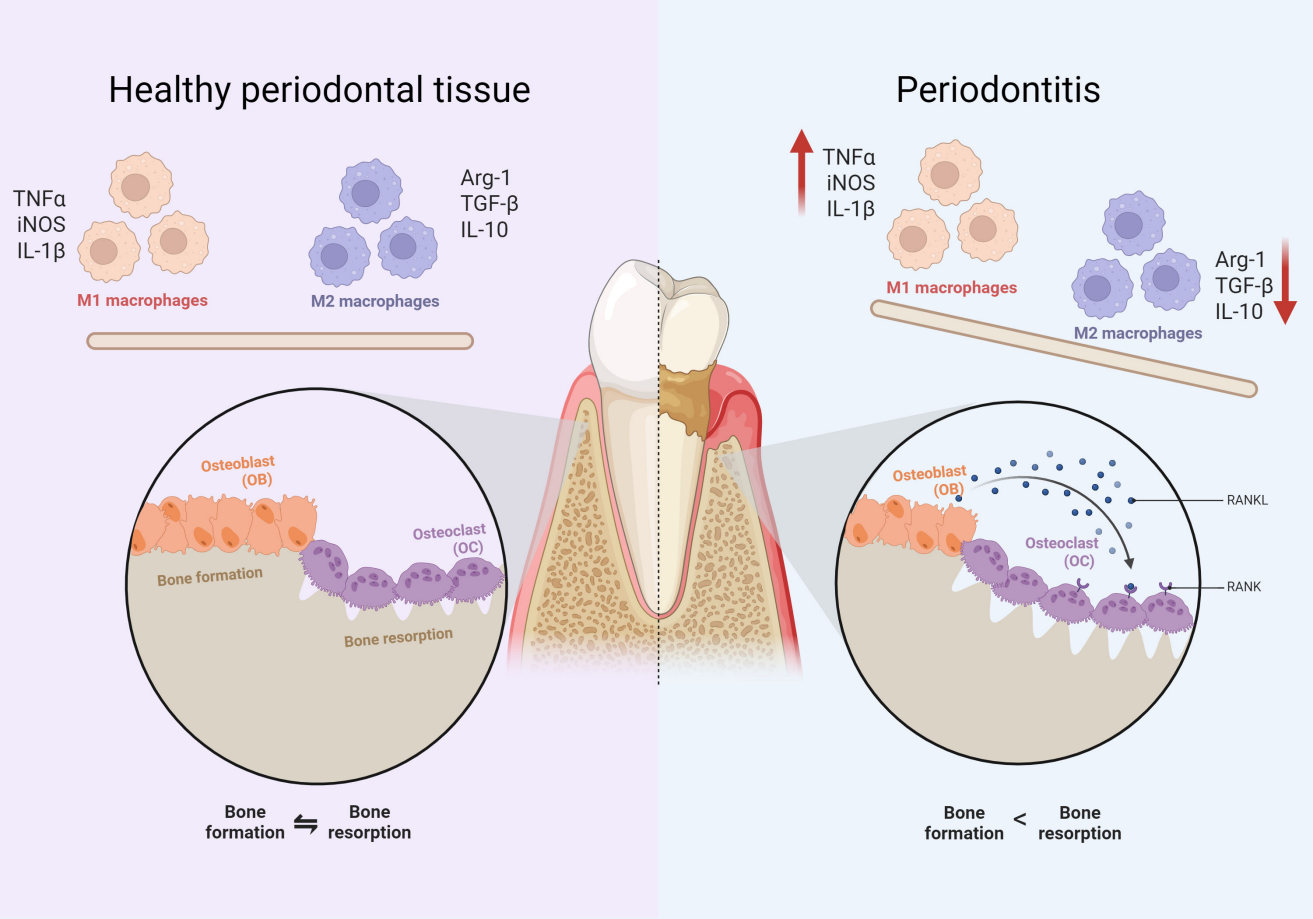
Comparison of cellular components between periodontitis and normal tissues. The left side indicates healthy periodontal tissue and the right side indicates periodontitis. It can be found that compared to healthy periodontal tissue, the proportion of M1 macrophages is up-regulated. The proportion of M2 macrophages decreases in periodontitis conditions. There is an imbalance between osteoblasts and osteoclasts of the alveolar bone, manifested by greater bone resorption than bone formation.

It has been demonstrated that periodontitis is often accompanied by an imbalance in the periodontal immune microenvironment. The periodontal immune microenvironment consists of a variety of cells, extracellular matrix and various cytokines, which interact with each other and form a complex network (Han et al., 2023). Macrophages are important immune cells involved in the immune microenvironment of periodontitis (**Figure 1**). In inflammatory periodontal sites, macrophages account for approximately 5-30% of infiltrating cells and play an important role in participating in the inflammatory response, and bone loss in periodontitis (Wang et al., 2020a). In addition, macrophages assist in the process of acquired immunity. Macrophages present bacterial antigens to lymphocytes, which activate an adaptive immune response and recruit other immune-inflammatory cells to the affected site (Yang et al., 2018; Locati et al., 2020).

Based on their function in inflammation and host defense, macrophages can be classified as classically activated macrophages and alternatively activated macrophages (also known as M1 and M2, respectively). Early changes in periodontitis are predominantly associated with up-regulation of the M1 phenotype, with late inflammatory regression mediated by Th2, Treg lymphocytes and M2 macrophages (Alghamdi et al., 2023). Exogenous PAMPs, such as LPS released by bacterial lysis, lead to macrophage M1 polarization and release of large quantities of pro-inflammatory cytokines, including TGF-α and PGE2, as well as proteases, such as matrix metalloproteinase-1 (MMP1), which are key components of the inflammatory cascade response mediating soft tissue degradation in periodontal disease and destruction of alveolar bone. M2 macrophages secrete anti-inflammatory cytokines that contribute to tissue repair and inflammation suppression, promote tissue regeneration, and restore homeostasis in periodontal tissues (Sloniak et al., 2023). Further, some researchers have also subdivided M2 macrophages into M2a, M2b and M2c (Martinez et al., 2008). However, some researchers have also proposed that considering the complexity of the *in vivo* microenvironment, the concepts of M1 and M2 are not sufficient to account for the diversity of macrophages, and therefore macrophages are reclassified based on the functions of different types of macrophages and the molecules on their surface (Ishida et al., 2023). In addition, periodontitis is also an inflammatory osteolytic disease, imbalance between osteoblasts and osteoclasts is an important mechanism of bone loss in periodontitis (Lin et al., 2021) (**Figure 1**). Osteoclast differentiation of macrophages plays a key role in this process (Sima et al., 2019). Osteoclasts and macrophages share a common origin, both originating from the myeloid monocyte-macrophage system (Yang and Wan, 2019). Macrophages are induced to differentiate into osteoclasts by colony-stimulating factor (M-CSF) and NF-κB ligand activator (RANKL) (Yao et al., 2021). The former mainly acts on osteoclast precursor cells to promote their proliferation and survival, while the latter is essential for the maturation of pro-osteoclasts in multinucleated osteoclasts (Xing et al., 2012). It has been demonstrated that up-regulation of RANKL/RANK, a key pathway mediating macrophage osteoclast differentiation (Kittaka et al., 2023), ultimately activates several transcription factors in the setting of periodontitis, such as NFATc1 (Takayanagi, 2007). Activation of these factors in turn regulates osteoclast-specific genes, which are required for osteoclast differentiation.

Mitochondria, as dynamic organelles, serve as central hubs for cellular energy metabolism, reactive oxygen species (ROS) regulation, and calcium homeostasis (Chandel, 2014). In immune cells such as macrophages, mitochondrial metabolism is not only crucial for ATP production but also acts as a key modulator of functional phenotypes (O’Neill and Pearce, 2016). For instance, shifts in mitochondrial metabolic pathways (e.g., oxidative phosphorylation vs. glycolysis) directly influence macrophage polarization toward pro-inflammatory (M1) or anti-inflammatory (M2) states, while mitochondrial ROS (mtROS) generation serves as a signaling molecule for inflammasome activation.

In the context of periodontitis, emerging evidence indicates that dysregulation of these mitochondrial functions—driven by bacterial pathogens and host inflammatory responses—contributes significantly to immune imbalance and tissue destruction (Jiang et al., 2023b). Mitochondrial dysfunction is increasingly implicated in periodontitis pathogenesis. Clinical studies reveal altered mitochondrial dynamics in patients, such as downregulated MFN1 in gingival crevicular fluid (Kırmızıgül Ö et al., 2024). P. gingivalis LPS impairs mitochondrial bioenergetics by elevating ROS in gingival cells (Napa et al., 2017), while F. nucleatum and P. gingivalis suppress mitochondrial fusion genes (MFN1/2) in fibroblasts (Aral et al., 2021).

Recently, the role of mitochondrial metabolism as a coordinator of macrophage function has emerged as a pivotal research focus. Mitochondria are major energy generators and are involved in macrophage function. For example, mitochondria are involved in macrophage activation, polarization, and osteoclast differentiation through metabolic reprogramming, and oxidative stress (Misrani et al., 2021). Macrophage function is often accompanied by greater energy demands, which prompts changes in mitochondrial metabolism, such as the production of ROS via the mitochondrial electron transport chain, which is involved in macrophage signaling as well as killing pathogens (Starkov, 2008; Jin et al., 2024), and the fusion of mitochondrial biogenesis and fusion to maintain a stable number of mitochondria and to ensure the supply of energy to macrophages (Vásquez-Trincado et al., 2016). The disruption of the tricarboxylic acid cycle (TCA cycle) and consequently the production of metabolic intermediates may also regulate macrophage phenotype and function by modulating innate immune signaling pathways, leading to the production of cytokines, anti-microbial peptides or tissue repair factors (O’Neill et al., 2016).

More and more evidence suggests that mitochondrial dysfunction is involved in the pathogenesis of a variety of chronic inflammatory diseases, including neurodegenerative, cardiovascular, metabolic and autoimmune diseases (Orekhov et al., 2020). The understanding of the relationship between altered mitochondrial function and periodontitis is also deepening (Soliman Wadan et al., 2024). Recent evidence suggests that altered mitochondrial metabolic patterns, oxidative stress, altered levels of mitophagy and ontogeny, dynamic injury and mitochondrial detachment may contribute to macrophage function in periodontitis.

**Figure 2:**
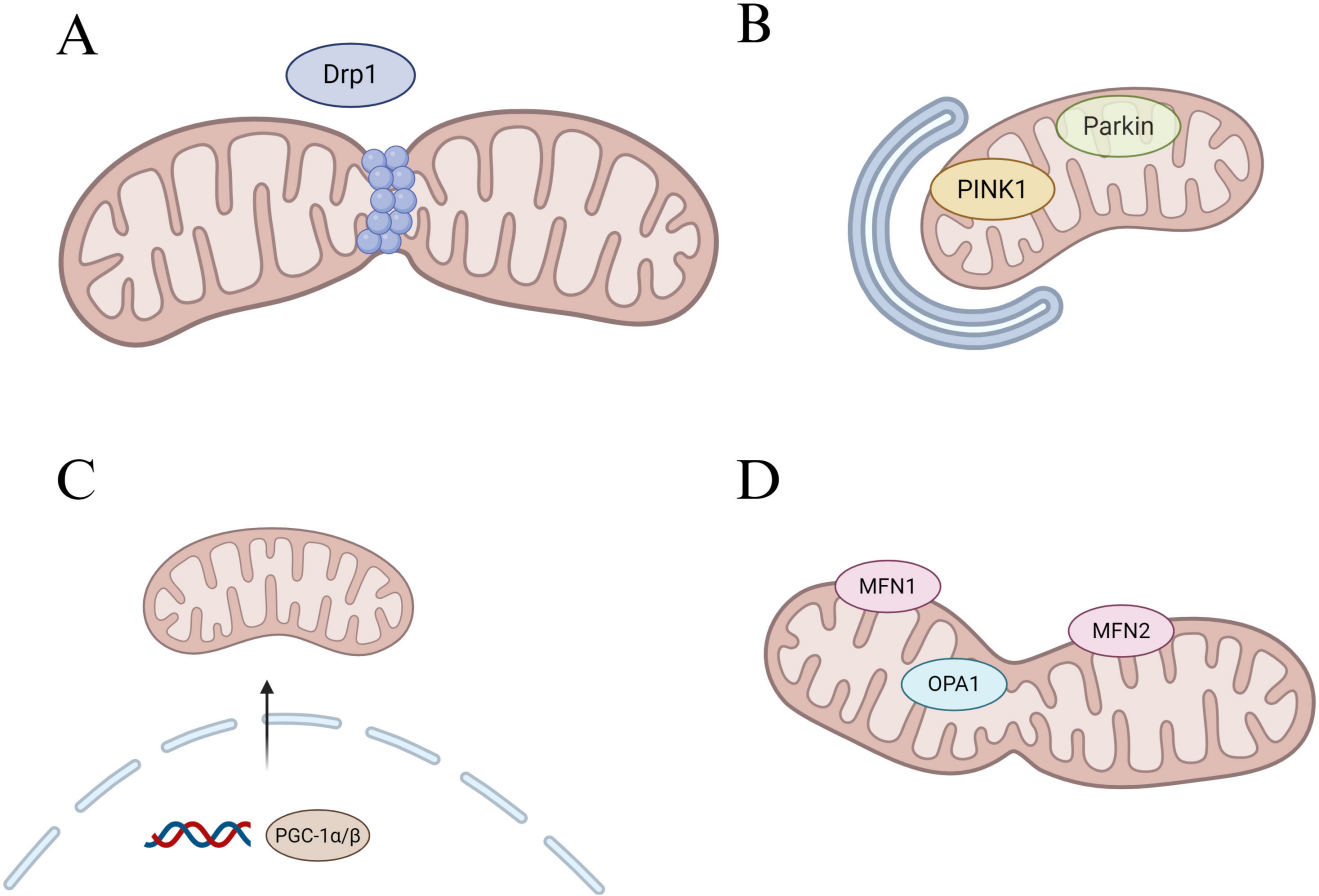
Regulation of mitochondrial core dynamic process. **(A)** Schematic diagram of mitochondrial fission, Drp1 is the main fission regulator. **(B)** Schematic diagram of mitophagy, PINK1 accumulates in the outer mitochondrial membrane (OMM) and recruits Parkin to the mitochondrial surface, which in turn triggers mitophagy. **(C)** Schematic diagram of mitochondrial genesis, PGC-1α/β acts as a general regulator of mitochondrial biogenesis by activating the transcription of downstream genes. **(D)** Schematic diagram of mitochondrial fusion, Mfn1 and Mfn2-mediated fusion of the OMM, and OPA1 control of the inner mitochondrial membrane fusion.

Therefore, in this review, we focus on the macrophage, a key immune cell that has been demonstrated in the pathogenesis of periodontitis, and discuss its mitochondrial dysfunction in the setting of periodontitis as well as the impact on its polarization and osteoclast differentiation functions. Moreover, we highlight promising periodontitis interventions targeting macrophage mitochondria that have shown therapeutic efficacy in preclinical models. It is hoped that this adopted review will further enrich the mechanisms of macrophage action in periodontitis and provide effective therapeutic strategies targeting mitochondria for periodontitis treatment (**Figure 2**).

## 2 Mitochondrial dysfunction affects macrophages in periodontitis

### 2.1 Metabolic reprogramming

Many immune cells undergo extensive metabolic pattern changes in the setting of periodontitis, resulting in a shift from oxidative phosphorylation (OXPHOS) to glycolysis, which is known as ‘metabolic reprogramming’ (Xia et al., 2021). It is currently believed that metabolic reprogramming is driven by the down-regulation of OXPHOS caused by mitochondrial dysfunction and the up-regulation of glycolysis caused by enhanced glycolytic pathways. Different mitochondrial metabolic patterns correlate with macrophage function. For example, aerobic glycolysis generates less ATP (only 2 ATP molecules per glucose molecule) but is favored in cells with high energy demands due to its advantage of faster production (Ganapathy-Kanniappan and Geschwind, 2013).

#### 2.1.1 macrophage polarization

Overall, M1 macrophages tend to utilize aerobic glycolysis to generate energy (in the form of four-molecule ATP), while M2 macrophages rely mainly on fatty acid oxidation (FAO) and glucose utilization through the TCA cycle to generate ATP (Dumont et al., 2021).

Periodontitis is often accompanied by impaired cellular mitochondrial function and reduced efficiency of the electron transport chain (ETC). Cells compensate for the lack of mitochondrial function by glycolysis, which supports the rapid proliferation and bactericidal activity of macrophages (Kelly and O’Neill, 2015). This increased glycolysis may be due to the up-regulation of the enzyme ubiquitin 6-phosphofructo-2-kinase/fructose-2,6-bisphosphatase (uPFK2), which induces high concentrations of fructose-2,6-bisphosphate, thereby enhancing glycolysis (Rodríguez-Prados et al., 2010). Interestingly, this glycolysis in M1 macrophages remains a major source of energy in an aerobic environment. Metabolic reprogramming toward glycolysis in M1 macrophages—characterized by increased glucose uptake and lactate production even in normoxia—serves as a rapid energy source for pro-inflammatory responses (Tannahill et al., 2013a) (Baseler et al., 2016). In periodontitis, this shift is triggered by bacterial LPS and inflammatory cytokines, as observed in salivary metabolomic analyses (Foratori-Junior et al., 2023). There were some individuals with severe periodontitis in the experiment who had low concentrations of lactate and high levels of acetic and propionic acid in their saliva. This may be related to the altered composition of the oral microbiota, considering that it has been demonstrated that lactic acid can be metabolized to acetic and propionic acids by bacterial species such as Haemophilus parainfluenzae, Lactobacillus casei, Fusobacterium, Propionibacterium, Trichosporon, and Verrucomicrobium (Liebsch et al., 2019).

Baseler et al. demonstrated that autocrine IL-10 fine-tunes M1 macrophage glycolytic commitment by modulating nitric oxide production, revealing a critical rheostat mechanism in inflammatory metabolic reprogramming (Baseler et al., 2016). In periodontitis, Zhu et al. reported that PKM2-mediated glycolytic reprogramming in bone marrow stromal cells exacerbates osteogenic dysfunction under diabetic conditions, highlighting the context-specific metabolic shifts in periodontal tissues (Zhu et al., 2025).

**Figure 3:**
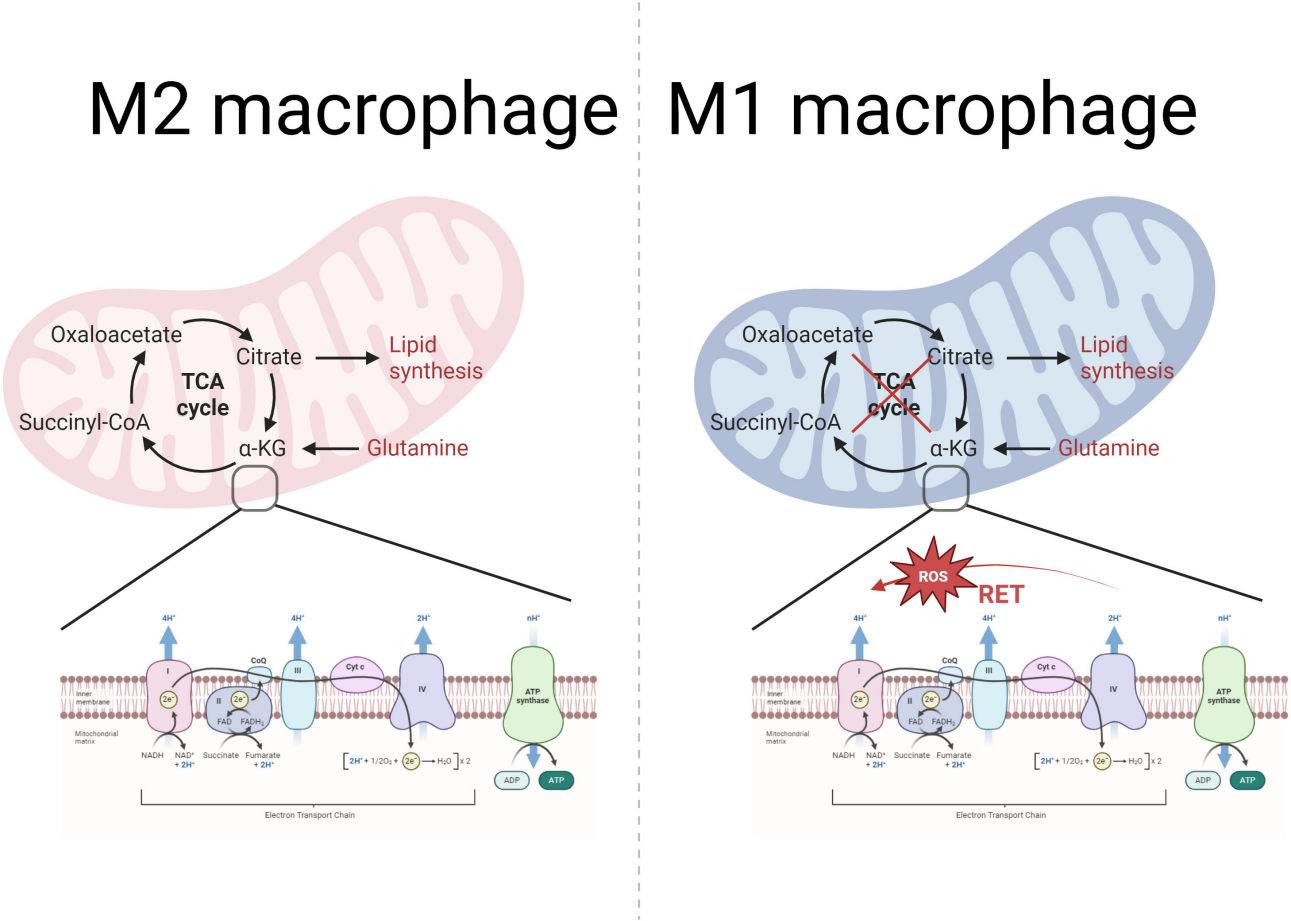
Mitochondrial metabolism in M2 and M1 macrophages: TCA cycle and OXPHOS. The left part represents the TCA cycle in the mitochondria of M2 macrophages with the mtETC-OXPHOS system. As the figure shows, the TCA cycle is normal in M2 macrophages. The mtETC-OXPHOS system exists in the inner membrane of the mitochondria, and the OXPHOS system consists of four complexes, ATP synthase and two mobile electron carriers (CoQ and Cytc). Among them, complexes I, III and IV have a proton pump function and are responsible for pumping protons from the mitochondrial matrix to the membrane interstitial space to form a proton gradient. While complex II is not involved in proton pumping and only transfers electrons to CoQ. ATP synthase uses the proton motive force potential generated by the ETC to pump protons from the membrane gap back to the mitochondrial matrix, while phosphorylating ADP to ATP, which provides energy to the cell. In contrast, right-sided M1 macrophages have impaired TCA cycling, which in turn affects mtETC efficiency and reduces oxidative phosphorylation. In addition, M1 macrophages showed reverse electron transfer (RET), resulting in increased electron leakage and increased mtROS production.

In addition, TCA cycle disruption accompanied by mitochondrial oxidative phosphorylation dysfunction in M1 macrophages is also considered to be involved in metabolic reprogramming. In M2 macrophages, the TCA cycle is intact and NADH and FADH_2_ produced during the process provide electron donors for oxidative phosphorylation. In contrast, in M1 macrophages, the TCA cycle is broken at both citrate and succinate, which in turn leads to impaired oxidative phosphorylation, resulting in reduced production of NADH and FADH_2_ with accumulation of intermediates, which in turn affects the efficiency of the mETC and reduces oxidative phosphorylation (Kumar et al., 2024) (**Figure 3**). In addition, in M1 macrophages, iNOS activity is up-regulated, leading to increased production of NO. NO is able to nitrate Fe-S proteins in CI and CIV leading to their inactivation, thus decreasing OXPHOS (Fernando et al., 2019). A negative correlation has been found between high-dose vitamin A intake and the likelihood of periodontal disease (Mi et al., 2024). Cheng et al. found that vitamin A was able to regulate the mitochondrial metabolic reprogramming of macrophages through the JAK-STAT signaling pathway, partially reversing LPS-induced mitochondrial damage and restoring macrophage oxidative phosphorylation, thereby inhibiting the progression of periodontitis (Cheng et al., 2025).

Recent studies have revealed that genetic mutations, microenvironmental alterations, and epigenetic modifications are also involved in the generation of the Warburg effect (Jing et al., 2020). Cameron et al. demonstrated that LPS-stimulated macrophages increase their nicotinamide-phosphate ribosyltransferase (NAMPT) expression, and that this increase in NAMPT expression permits the maintenance of a sufficient pool of NAD+ to sustain glyceraldehyde-3-phosphate dehydrogenase activity and Warburg metabolism (Cameron et al., 2019).

The different metabolic characteristics of M1/M2 macrophages are adapted to their function. It has been shown that NaF promotes glycolysis and M1 polarization in macrophage, thereby exacerbating periodontitis (Bi et al., 2024). Zhang et al. used quercetin to inhibit macrophage aerobic glycolysis, reprogramming inflammatory macrophages to an anti-inflammatory phenotype, and attenuating the symptoms of periodontitis and periodontal tissue destruction (Zhang et al., 2024). Targeting macrophage aerobic glycolysis provides a new strategy for periodontitis treatment.

#### 2.1.2 Effect of mtROS on macrophage polarization

**Figure 4:**
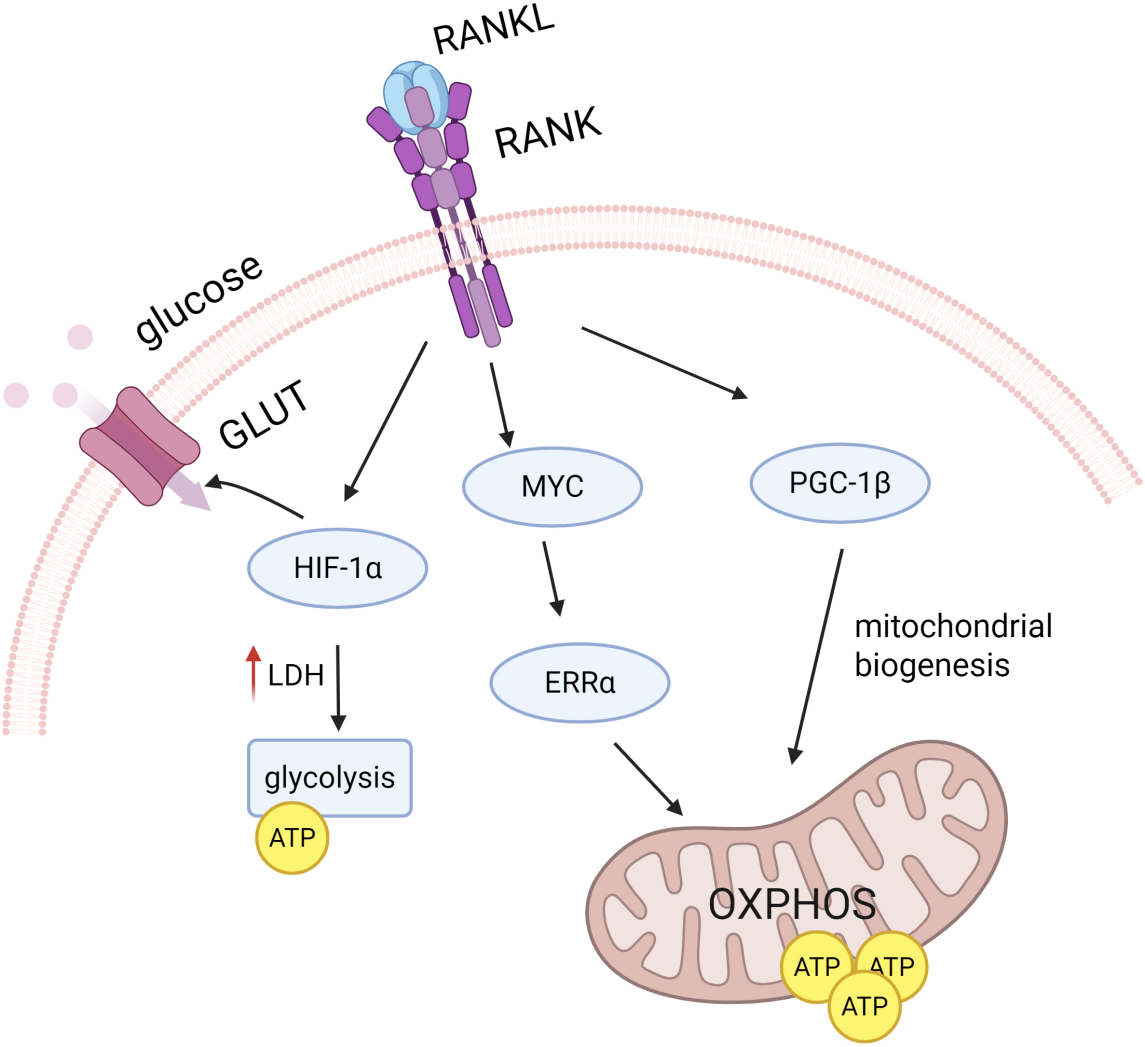
RANKL signaling regulates osteoclast differentiation. RANKL signaling promotes OXPHOS and ATP production by inducing PGC1β, which is involved in mitochondrial biogenesis, and the MYC-ERRα axis. RANKL also increases GLUT number and glycolysis by up-regulating HIF-1α during the later stages of osteoclast differentiation, thereby increasing glucose uptake and utilization.

The metabolic patterns of macrophages vary at different stages of osteoclast differentiation. RANKL increases mitochondriogenesis during osteoclast differentiation and promotes OXPHOS and ATP production. During the final stages of osteoclast differentiation, RANKL also increases glycolysis and lactate synthesis to meet the high energy demands of bone resorption (Marques-Carvalho et al., 2024) (**Figure 4**). It has been demonstrated that OXPHOS is up-regulated in osteoclasts, providing an energy supply especially during the early stages of osteoclast differentiation (Park-Min, 2019). By blocking the ETC using a mitochondrial complex I inhibitor, the OXPHOS process is blocked and macrophages will be unable to differentiate into osteoclasts (Jin et al., 2014).

Up-regulation of OXPHOS may be associated with mitochondrial biogenesis during osteoclast differentiation. Several studies have used OXPHOS as a measure of the level of mitochondrial biogenesis (Zeng et al., 2015). In recent years, pathways independent of mitochondrial biogenesis have also been reported. MYC, an important transcription factor induced by RANKL, has been proven to drive the regulation of macrophage osteoclast differentiation by OXPHOS through the MYC/ERRα pathway. It was found that overexpression of MYC failed to regulate PGC1β expression, suggesting that the MYC/ERRα pathway may regulate OXPHOS in osteoclasts independently of mitochondrial process (Bae et al., 2017).

Glucose has been identified as the main energy source for osteoclasts during late osteoclast differentiation. During osteoclastogenesis, glucose uptake increases significantly. The glucose transporter protein GLUT3 is predominantly expressed in osteoclast precursors, whereas GLUT1 is up-regulated in mature osteoclasts (Da et al., 2021). Ahn et al. found that RANKL-induced osteoclast differentiation exhibits increased lactate dehydrogenase (LDH) activity, enhanced glycolysis, and promotes the formation of mature osteoclasts via the NFATc1 signaling pathway (Ahn et al., 2016). Similarly, Li et al. significantly reduced osteoclast production by inhibiting the key enzyme pyruvate kinase M2 (PKM2) in glycolysis and pyruvate metabolism (Li et al., 2024a). In addition, during osteoclastogenesis, RANKL is able to activate HIF-1α and induce the expression of GLUT1 and glycolytic enzymes (Indo et al., 2013). Compared to OXPHOS, glycolysis via HIF-1α enables osteoclasts to rapidly increase ATP production under hypoxic conditions, which results in more efficient osteoclast function (Morten et al., 2013).

### 2.2 mitochondrial ROS release

Oxidative stress refers to the disruption of the balance between oxidative and antioxidant systems, resulting in the overproduction of ROS, which comprise a variety of types including O2-, H2O2, HO- and NO (Pospíšil et al., 2019). Mitochondria, as energy-producing organelles, produce ROS as a by-product of the ETC, but only 1-3% of ROS are produced under normal conditions due to the presence of antioxidant systems (e.g., the glutathione system, superoxide dismutase, coenzyme Q10, etc.) (Zorova et al., 2018). In activated macrophages, NADPH oxidase and mitochondrial ETC activity are thought to be the main sources of ROS (Hall et al., 2013). Suitable concentrations of ROS are able to defend against microbial invasion and also act as second messengers with positive biological effects, especially H2O2 (Averill-Bates, 2024).

Under some conditions, if antioxidant systems are unable to adequately counteract the deleterious effects of elevated ROS, cellular damage can occur. Excessive accumulation of ROS accelerates the progression of periodontitis, including increased periodontal tissue damage and alveolar bone loss (Sczepanik et al., 2020). The association between oxidative stress and periodontitis is further strengthened by increasing evidence of elevated levels of oxidative stress markers in saliva, gingival sulcus fluid and plasma of patients with periodontitis (Önder et al., 2017; Gurbuz et al., 2024).

mtROS release is involved in periodontal inflammatory progression and tissue destruction. Studies have demonstrated that increased mitochondrial fission leads to excessive ROS production, and dynamin-related protein 1 (Drp1) is an important regulator of mitochondrial fission (Wu et al., 2024). Shi et al. found that in a periodontitis model, Drp1 expression was significantly higher than that of the control group, while blocking Drp1 resulted in a decrease in ROS levels and suppression of periodontal cell apoptosis and inflammatory responses. This demonstrates the role of Drp1-ROS in the pathogenesis of periodontitis (Shi et al., 2021b). In addition, West et al. revealed that bacterial-induced activation of TLR1/2/4 signaling may contribute to macrophage mtROS release. Activation of this pathway involves translocation of the Toll signaling adaptor tumor necrosis factor receptor-associated factor 6 (TRAF6) to mitochondria and causes ubiquitination of Ecsit (West et al., 2011). Ecsit is involved in the assembly and stabilization of mitochondrial complex I. Its ubiquitination interferes with mitochondrial oxidative phosphorylation processes, thereby increasing mtROS production (Vogel et al., 2007). In the setting of periodontitis, TLR4 is recognized as a key receptor for the activation of innate immunity by *Porphyromonas gingivalis* lipopolysaccharide (PgLPS) (Nativel et al., 2017). After recognition of bacterial surface lipopolysaccharide by Mφ via TLR4, the production of mtROS is increased through a signaling pathway involving TRAF6, thereby increasing macrophage killing of pathogenic bacteria (Cui et al., 2023). TRAF6 also activates the NF-κB and MAPK pathways to induce the release of large amounts of pro-inflammatory factors (TNF-α, IL-6, IL-1β), further amplifying the inflammatory response

The following section will focus on the effect of mtROS release on macrophage function.

#### 2.2.1 mtROS and macrophage polarization

The mtETC flows in different directions in M1/M2 type macrophages, leading to mtROS production in M1 macrophages. In M2 macrophages, electron flow is ordered in a positive manner, which leads to reduced electron leakage and low final mtROS production (Kumar et al., 2024). In contrast, reverse electron transfer (RET) is present in M1 macrophages (**Figure 3**). RC-I is the first component of the ETC, transferring electrons from NADH to coenzyme Q via several iron-sulfur clusters (Ojha et al., 2022). Complex II (CII) acts to catalyze the oxidation of succinate to fumarate while transferring electrons to ubiquinone (Sousa et al., 2018). However, when the proton gradient is too high or in the presence of a high concentration of succinate, CII continuously injects electrons into the ubiquinone pool, and this enrichment of UQH_2_ leads to a reverse transfer of electrons to CI, resulting in CI being a major source of ROS (Adam-Vizi and Chinopoulos, 2006). In addition, Dubouchaud et al. demonstrated that RET may also be related to the NADH/NAD + ratio (Dubouchaud et al., 2018).

Due to impaired TCA cycling, succinate is often enriched in M1 macrophages, generating mtROS via RET. Mills et al. demonstrated that in LPS-stimulated macrophages, increased mitochondrial oxidation of succinate via SDH and elevated ΔΨm collectively facilitated the production of RET and subsequent mtROS in complex I. Furthermore, increased mtROS levels induced increased macrophage IL-1β expression. Using rotenone, the complex I inhibitor, was effective in decreasing IL-1β mRNA expression, supporting the role of mtROS species production for macrophage polarization in inflammation (Mills et al., 2016).

Cytochrome c oxidase (CcO) is the terminal oxidase of the mitochondrial ETC that catalyzes the transfer of electrons from reduced cytochrome c to molecular oxygen (Srinivasan and Avadhani, 2012). Angireddy et al. showed that cytochrome c oxidase dysfunction leads to increased release of mtROS, which induces macrophages to produce the pro-inflammatory cytokines (Angireddy et al., 2019).

**Figure 5:**
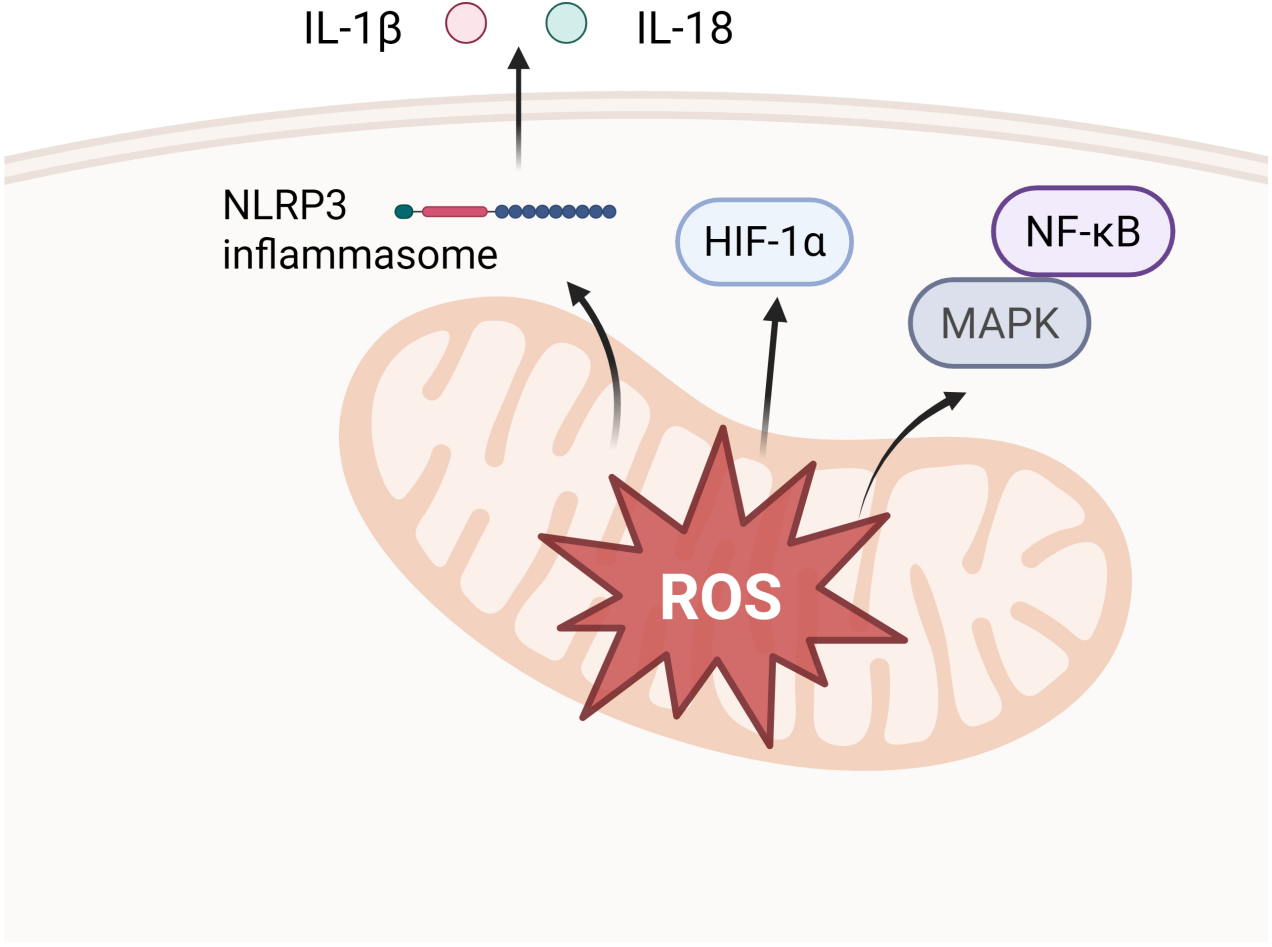
Downstream pathways of mtROS release. Activation of MAPK and NF-κB pathways (e.g. activation of p38 into the nucleus, up-regulation of IκBα transcription, etc.), which in turn elicits an inflammatory response. Stabilization of HIF-1α, which facilitates aerobic glycolysis and IL-1β induction in LPS-activated macrophages. Activation of the NLRP3 inflammatory vesicle, activation of Caspase-1, cleavage of pro-IL-1β and pro-IL-18 to transform them to active forms.

Mechanistically, mtROS may be involved in macrophage M1 polarization in periodontitis through activation of MAPK and NF-κB pathways, activation of NLRP3 inflammasome and stabilization of HIF-1α (**Figure 5**).

##### 2.2.1.1 Activation of MAPK and NF-κB signaling pathways

mtROS can act as pro-inflammatory signals, inducing pro-inflammatory gene expression and macrophage polarization in the M1 direction by the MAPK and NF-κB pathways (Liu et al., 2024b). The MAPK pathway signals through a cascade reaction of the MAPK family of kinases and performs different functions through four major branches, JNK, p38/MAPK, ERK and ERK5. Among them, activation of the p38/MAPK pathway was shown to be possibly associated with mtROS, which is able to promote macrophage M1 polarization. Protein levels of phospho-p38 MAPK (pp38) were found to be elevated in macrophages after LPS/ATP stimulation, whereas inhibition of mtROS activity with rapamycin resulted in down-regulation of pp38 levels and inhibition of suppression of IL-6 and IL-8 transcription (Ko et al., 2017). As for the activation of the NF-κB pathway by mtROS, mtROS phosphorylates IκB kinase (IKK), leading to the degradation of IκB, which in turn releases NF-κB. Upon entry into the nucleus of the cell, NF-κB induces the gene expression of several inflammatory factors such as IL-1β, IL-6 and TNF-α, initiating the inflammatory response and driving macrophages to M1 polarization (Sun et al., 2021; Zhou et al., 2022). Herb et al. demonstrated that mtROS induces covalent intermolecular attachment of κB (IκB) kinase (IKK) complex regulatory subunit NEMO through disulfide bonds formed by Cys54 and Cys347, which activates the IKK complex and subsequent signaling via the ERK1/2 and NF-κB pathways, ultimately leading to the secretion of pro-inflammatory cytokines (Herb et al., 2019).

##### 2.2.1.2 Activation of the NLRP3 inflammasome

Increasing evidence confirms that mtROS is an important molecule for NLRP3 inflammasome activation, and that LPS-induced mitochondrial dysfunction increases mtROS production, which further causes NLRP3 deubiquitination (Zhang et al., 2022b). Under LPS-induced conditions, H2 inhibited NLRP3 inflammasome activation by inhibiting mtROS-mediated NLRP3 deubiquitination (Ren et al., 2016), suggesting an mtROS-dependent activation of NLRP3 inflammasome.

NLRP3 inflammasome activation is associated with M1/M2 macrophage imbalance, which is a driver of a number of inflammatory diseases including periodontitis. NLRP3 inflammasome activation depends on two functionally distinct steps: ‘priming’ and ‘activation’ (Zhong et al., 2018). Priming relies on the direct engagement of Toll-like receptors via pathogen-associated or injury-associated molecular patterns, leading to the rapid activation of NF-κB, which stimulates the synthesis of IL-1β precursors and increases the expression of NLRP3. Activation, that is, inducing a specific form of mitochondrial damage in the presence of NLRP3 activators. The release of fragmented mtDNA and increased production of ROS promotes the assembly of NLRP3 inflammasome and mediates Caspase-1 activation. Caspase-1 shears precursor forms of IL-1β and IL-18, which eventually mature and are released (Paik et al., 2021).

Inhibition of NLRP3 inflammasome initiation or activation interferes with macrophage M1 polarization and inflammatory cytokine release. Inhibition of NLRP3 inflammasome activation in macrophages using 1,8-cineole effectively reduced macrophage expression levels of IL-1β, TNF-α, IL-6, IL-18 and IL-23, inhibited M1 polarization and improved the inflammatory environment (Ma et al., 2023). Zhang found that the production levels of NLRP3, caspase-1, pro-caspase-1 and IL-1β were up-regulated in M1 macrophages *in vitro* experiments (Zhang et al., 2020). Yang et al. observed that the expression of NLRP3, GSDMD, cleaved-IL-1β and cleaved-caspase-1 was up-regulated in LPS-treated RAW264.7 cells and in a murine periodontitis model (Yang et al., 2024). Clinical experiments have supported the idea that salivary levels of NLRP3 and IL-1β can be used as a measure of the extent of periodontal tissue destruction (Mitra et al., 2022; Abraham et al., 2023), suggesting that NLRP3 inflammasome activation induced by an imbalance of M1/M2 macrophages may be involved in the pathogenesis of periodontitis.

##### 2.2.1.3 HIF-1α stabilization

HIF-1α is hydroxylated by prolyl hydroxylase (PHD) under normoxic conditions and then degraded via the ubiquitin-proteasome pathway (Chandel et al., 2000). It has been shown that mtROS stabilizes HIF-1α, thereby promoting aerobic glycolysis and IL-1β induction in LPS-activated macrophages (Wang et al., 2021). To elucidate the effect of mitochondrial complex I-dependent ROS on HIF-1α after LPS stimulation, Fuhrmann et al. designed THP-1 macrophages knocking down the transmembrane protein (TMEM126B) (Fuhrmann et al., 2019). TMEM126B is a complex I assembly factor, and defects in TMEM126B cause complex I defects associated with mtROS production (Heide et al., 2012). The results indicated that TMEM126B-deficient macrophages had diminished HIF-1α stability and reduced IL-1β expression even after LPS stimulation (Fuhrmann et al., 2019). Similarly, Mills et al. demonstrated that mtROS produced via RET activates HIF-1α, which causes an increase in macrophage production of the pro-inflammatory cytokine IL-1β (Mills et al., 2016).

#### 2.2.2 mtROS and macrophage osteoclast differentiation

Binding of RANK to RANKL leads to rapid degradation of IκBα and release and nuclear translocation of p65 protein from the NF-κB protein complex. It plays a central role in osteoclast formation, activation and regulation of bone metabolism (Chen et al., 2024). There is growing evidence that RANK-RANKL signaling forms a positive feedback with ROS generation and promotes osteoclast differentiation (Yang et al., 2004; Lee et al., 2005). Although the NADPH oxidase system (Nox1 and Nox2) is a major contributor to ROS-mediated bone resorption, mtROS production has also been shown to play an important role in osteoclast differentiation and function (Ha et al., 2004; Srinivasan and Avadhani, 2007). Experiments demonstrated that mtROS and MMP levels were significantly increased in RAW264.7 cells exposed to RANKL compared to controls, suggesting that mtROS are associated with the process of macrophage osteoclast differentiation (Jiang et al., 2024). In another *in vitro* experiment, Srinivasan et al. identified the production of mtROS due to respiratory stress under hypoxic conditions as an important contributor to osteoclast differentiation, and MitoQ (0.35 µM) effectively prevented the formation of TRAP-positive multinucleated cells (Srinivasan et al., 2010).

Mechanistically, the mtROS-mediated osteoclast differentiation of macrophages may be mediated by the Ca2+/Calcineurin-NFATc1 pathway. As a result of mitochondrial stress, mtROS increases, causing a burst release of mitochondrial Ca2+ ions into the cytoplasm causing calmodulin phosphatase-mediated inactivation of IκBβ and activation of NF κ B/Rel factor, which ultimately leads to an increase in the level of NFATc in the nucleus (Biswas et al., 2003).

### 2.3 Mitochondrial calcium dysregulation

Mitochondria serve not only as cellular powerhouses but also as critical calcium buffers, playing a vital role in maintaining intracellular calcium homeostasis (Rizzuto et al., 2012). Mitochondrial dysfunction impairs calcium uptake and storage capacity, leading to pathological elevation of cytosolic calcium (calcium overload). This overload disrupts mitochondrial membrane potential (ΔΨm) and triggers irreversible opening of the mitochondrial permeability transition pore (mPTP) (Baines, 2009; Marchi et al., 2018). Sustained mPTP opening causes mitochondrial swelling, outer membrane rupture, and release of pro-apoptotic factors (e.g., cytochrome *c*), ultimately activating caspase cascades and inducing apoptosis (Giorgi et al., 2018). In the inflammatory milieu of periodontitis, immune cells such as macrophages endure persistent stressors (e.g., pathogen LPS). Notably, P. gingivalis LPS has been shown to induce calcium overload and mitochondrial damage in PDLC (Liu et al., 2022a). Impaired mitochondrial calcium handling in these cells may exacerbate death pathways, hinder inflammation resolution, and compromise tissue repair, thereby driving progressive periodontal destruction.

### 2.4 Down-regulation of mitophagy

Mitophagy is a selective autophagic process in which autophagosomes are formed to encapsulate damaged mitochondria in response to external stimuli, such as reactive ROS, nutrient deficiency, and cellular senescence. And then autophagosomes fuse with lysosomes, thereby removing dysfunctional mitochondria and maintaining cellular stability (Onishi et al., 2021). Under stressful or inflammatory conditions, mitophagy prevents the accumulation of damaged mitochondria and the increase of ROS homeostatic levels, which would otherwise lead to oxidative stress and cell death (Wang et al., 2021). The process of mitophagy can be carried out by both ubiquitination and non-ubiquitination pathways. The former is mediated by the PINK1-Parkin pathway and the latter through mitochondrial receptors (Palikaras et al., 2018).

PTEN-induced putative kinase 1 (PINK1) is an autophagy-related gene that mediates the most classical mitophagy. Defective mitochondria have a reduced mitochondrial membrane potential (Δ μ m) and enhanced fragmentation. At this time, PINK1 is unable to enter the inner mitochondrial membrane but instead accumulates in the outer mitochondrial membrane (OMM). This is a key step in the recruitment of Parkin to the mitochondrial surface, which further triggers mitophagy (Jiang et al., 2023b). Jiang et al.’s clinical trial revealed that the expression of PINK1, Parkin, and microtubule-associated protein light chain 3 (LC3) was significantly down-regulated in clinical specimens of gingival tissues from patients with periodontitis compared to those with healthy gingival tissues, suggesting that aberrant mitophagy is relevant to periodontitis (Jiang et al., 2023a).

To determine whether PINK1 contributes to the regulation of periodontitis-associated osteoclastogenesis and alveolar bone resorption, Jang performed ligation-induced periodontitis (LIP) on PINK1 knockout (KO) mice. The results revealed that PINK1 KO mice had lower bone volume fractions compared to wild-type mice. The number of TRAP-positive osteoclasts was significantly increased, suggesting that PINK1 is essential in maintaining mitochondrial homeostatic osteoclast differentiation (Jang et al., 2024).

To further investigate the mechanism by which PINK1 affects osteoclast differentiation, the researchers treated osteoclasts with PINK1 defects with spermidine (SPD) and n -acetylcysteine (NAC); respectively. SPD, is a natural polyamine that induces mitophagy and autophagy, and in the experiment was able to effectively attenuate the effects of PINK1 defects on osteoclast formation and the expression of the downstream target NFATc1 gene expression, thereby inhibiting excessive osteoclast differentiation. The researchers measured mtROS levels and found that mtROS levels were significantly increased in PINK1 KO cells compared to controls. In addition, treatment with NAC reduced osteoclast overproduction induced by PINK1 deficiency, thereby delaying periodontitis progression (Jang et al., 2024). This suggests that PINK1 is able to maintain normal mitochondrial morphology, stabilize mitochondrial membrane potential and reduce mtROS production, thereby inhibiting the overproduction of periodontitis osteoclasts in periodontitis.

Furthermore, researchers have discovered that inhibition of mitophagy triggers classical macrophage activation in a mtROS-dependent manner. Mechanistically, Patoli et al. demonstrated that inhibition of mitophagy is an early feature of macrophage activation. LPS activates inflammatory cysteine-aspartic enzymes1 and 11 via STAT1-dependent activation, which in turn inhibits PINK1-dependent mitophagy in macrophages (Patoli et al., 2020). In addition, Zhu et al. found that STAT3 signaling regulates macrophage NLRP3 inflammasome activation by inhibiting the PINK1-dependent mitophagy pathway, eliminating dysfunctional mitochondria, and inhibiting the release of mtROS, which in turn induces IL-1β release. In addition, performing STAT3 inhibition effectively protected mice from infection-induced periapical lesions. STAT3 inhibition was effective in inducing PINK1-dependent mitophagy, inactivating the NLRP3 inflammasome, and inhibiting macrophage infiltration and osteoclast formation (Zhu et al., 2023). This result suggests an important role for the STAT3/mitophagy axis in regulating macrophage function in bone loss disease.

### 2.5 Abnormal mitochondrial biogenesis

Mitochondria are semi-autonomous organelles. A small proportion of mitochondrial proteins associated with the respiratory chain are encoded by mtDNA, whereas the bulk of the mitochondrial proteome is encoded by the nucleus and is synthesized and translocated into the mitochondria (Moulin et al., 2019). Thus transcription and translation of nuclear and mitochondrial genes must be tightly coordinated to ensure mitochondrial biogenesis.

Mitochondrial biosynthesis is controlled by PPARγ coactivator-1 α (PGC-1α) and PGC-1β signaling pathways. Nuclear respiratory factors 1 (NRF1) and 2 (NRF2) encoded by the nucleus along with PGC-1α/β are important regulators of mitochondrial biogenesis (Bueno et al., 2020; Liu et al., 2023a). PGC-1α/β, as a general regulator of mitochondrial biogenesis, promotes mitochondrial biogenesis through downstream activation of NRF1 and NRF2, which trigger mitochondrial transcription factor A (TFAM) (Popov, 2020). In addition, Nrf2 has a role in regulating the expression of various antioxidant genes, thereby scavenging excess ROS to maintain mitochondrial homeostasis (Liu et al., 2021).

In recent studies, it has been found that periodontitis is often accompanied by abnormal mitochondrial biogenesis. Wang et al. discovered that the expression of PGC-1α and Nrf2 was significantly reduced in refractory apical periodontitis (Wang et al., 2024). Mitochondrial biogenesis and function are dependent on the translocase of the mitochondrial outer membrane (TOM) complex, of which subunit 20 (TOMM20) is the major receptor component (Tang et al., 2023). It facilitates the initial transport of proteins into mitochondria. However, the number of PGC-1α-TOMM20 double-positive cells was significantly lower in the RAP group than in the control group, suggesting the presence of dysfunctional mitochondrial biosynthesis in periapical lesions (Wang et al., 2024). Similarly, Sun et al. demonstrated that PGC-1α expression levels in periodontal tissues of periodontitis rats were lower than those of control rats, further demonstrating that periodontitis is accompanied by impaired mitochondrial biosynthesis (Sun et al., 2017).

Mitochondrial dysfunction in chronic periodontitis is confirmed, however, the correlation between mitochondrial biogenesis and dental osteoclast mineralization is poorly reported. The critical role of the MAPK pathway for dental osteoclast mineralization has been demonstrated (Xia et al., 2024). It has been shown that PGC-1α regulates mitochondrial biogenesis and promotes the cementoblast mineralization. And impaired MAPK pathway often present in hypoxic conditions, which resulting in a downward revision of PGC-1α (Wang et al., 2022). Ckip-1 has been reported to be a molecular switch during macrophage polarization, and CKIP-1 expression is significantly induced in M1 macrophages and reduced in M2 macrophages (Chen et al., 2017). Huang et al. used Ckip-1 silencing to promote M2 macrophage polarization. This genetically modified M2 macrophage transferred the exosome Let-7f-5p to cementoblast, which then accelerated the mineralization of cementoblast by targeting Ckip-1 to activate peroxisome proliferator-activated PGC-1α-dependent mitochondrial biogenesis (Huang et al., 2024).

In addition, mitochondrial biogenesis has been shown to be significantly up-regulated in osteoclast differentiation. PGC-1β has been shown to be a regulator of mitochondrial biosynthesis in osteoclasts. PGC-1β-deficient mice exhibit increased bone mass with impaired osteoclast function (Wei et al., 2010). Mechanistically, cAMP-responsive element binding protein (CREB) induces PGC-1β transcription during osteoclast differentiation (Ishii et al., 2009) and the process may be associated with elevated ROS. ROS scavenging using NAC significantly inhibited PGC-1β expression as well as osteoclast differentiation (St-Pierre et al., 2006).

Furthermore, mitochondria-related iron metabolism promotes PGC-1β expression, which enhances osteoclast differentiation and bone resorption activity (Zhang et al., 2019). It has been shown that activation of the RANKL signaling pathway can increase transferrin receptor 1 (TfR1)-mediated iron uptake, thereby contributing to the activation of mitochondrial respiration (Balogh et al., 2018). Ishii et al. identified that CREB activated downstream of RANK and ITAM during osteoclastogenesis induced the transcription of PGC-1β, which in turn stimulated mitochondrial biogenesis, leading to an increase in iron demand. Iron uptake via up-regulated TfR1 protein was supplied to heme proteins and iron-sulfur clusters in mitochondria, leading to increased mitochondrial respiration and ROS production, which further accelerated the transcription of PGC-1β via CREB via a positive feedback mechanism (Ishii et al., 2009). Further, Li et al. also found that abnormal iron metabolism plays an important role in periodontitis-induced bone loss by increasing mitochondrial genesis, which is involved in osteoclastogenesis (Li et al., 2024b). Thus, adjusting iron metabolism and affecting mitochondrial biogenesis may provide implications for intervening in osteoclast function in periodontitis.

### 2.6 Mitochondrial dynamic damage

Mitochondrial dynamics include mitochondrial fusion and fission, both of which play irreplaceable roles in maintaining mitochondrial structure and function (Wang et al., 2023a). Mitochondrial fusion, that is, the fusion of the membrane and matrix between two mitochondria, involves the fusion of the outer mitochondrial membrane mediated by mitochondrial fusion proteins 1 and 2 (Mfn1 and Mfn2) and the fusion of the inner mitochondrial membrane controlled by optic atrophy 1 (OPA1) (Jiang et al., 2023b). As for mitochondrial fission, Drp1 is the major fission regulator and belongs to the family of initiating proteins, which are usually present in the cytoplasm as monomers or oligomers. When activated, Drp1 translocates to the mitochondrial surface and forms multimers, thereby promoting mitochondrial fission (Michalska et al., 2016). Drp1 activation is the result of co-regulation of phosphorylation and dephosphorylation at different sites. Phosphorylation starts at several sites, including S616 and S637. Phosphorylation at S637 restricts fission by blocking the recruitment of Drp1 to mitochondria, whereas phosphorylation at S616 facilitates fission by driving Drp1 mitochondrial translocation (Chang and Blackstone, 2010). Upon activation, Drp1 connects to the OMM by binding to the articulin surface through a variable structural domain (insertion fragment B) (Adebayo et al., 2021). However, since Drp1 lacks a structural domain that binds directly to membrane phospholipids, it requires the aid of bridging proteins, including Fis1, Mff, MiD49, and MiD51 to adhere to the mitochondrial surface, and thus participates in mitochondrial membrane rupture (Liu et al., 2024a). An imbalance between the two processes of mitochondrial fusion and fragmentation has been linked to a variety of diseases such as cancer, inflammation-related diseases, neurodegenerative diseases and cardiovascular diseases (Jin et al., 2021; Al Ojaimi et al., 2022).

The relationship between altered mitochondrial dynamics and macrophage polarization has also been demonstrated in periodontitis. Jiang et al. examined gingival tissues from patients with periodontitis and found that the expression of phosphorylated Drp1 at the serine position 616 (p-Drp1 [Ser616]) was significantly elevated in the gingival tissues of patients with periodontitis. Stimulation of RAW264.7 cells with Pg LPS and THP-1 macrophages with Pg resulted in unchanged levels of total Drp1, while Drp1 tetramer formation showed a significant increase. This suggests the presence of macrophage overactivation of Drp1 in the setting of periodontitis (Jiang et al., 2025). In addition, researchers have found that over-activated Drp1 not only induces mitochondrial fragmentation but also drives NLRP3 inflammasome activation and subsequent inflammatory factor release. This was caused by a direct interaction between Drp1 and HK1 that promotes excessive mPTP opening [159]. The result is consistent with previous findings that excessive mPTP opening disrupts mitochondrial morphology, leading to mtROS release and oxidation of mitochondrial DNA (Ox-mtDNA) with subsequent activation of NLRP3 inflammasome (Xian et al., 2022).

A recent study has shown that phosphorylation of Drp1 at the Ser616 site may be mediated by Stat2 (Hu et al., 2022). Yu et al. RNA-seq analyses showed that LPS promotes the expression of signal transducer and activator of Stat2 and Drp1, which promotes mitochondrial fragmentation (Yu et al., 2020). The fragmented mitochondrial ETC is impaired, shifting its function from ATP synthesis to ROS production, which drives NFκB-dependent inflammatory cytokine transcription (Yu et al., 2020). This suggests a role for Stat2-Drp1 in macrophage M1 polarization.

Ma et al. demonstrated that lignocaine modulates mitochondrial dynamics to achieve macrophage M2 polarization and effectively ameliorates periodontitis. LUT was able to enhance mitochondrial fusion as evidenced by increased expression of macrophage fusion-associated proteins (e.g., MFN1 and MFN2) in periodontitis-simulated environments *in vitro*. In addition, LUT inhibited mitochondrial fission by downregulating DRP1. Alterations in these key proteins enhanced mitochondrial activity in macrophages and stimulated M2 polarization (Ma et al., 2025).

Osteoclasts are the product of cell fusion, and mitochondrial fusion helps to maintain mitochondrial membrane potential and respiratory chain function, thereby supporting the high metabolic demand of osteoclasts (Ribeiro et al., 2023). Previous studies have shown that AMPK can act as an energy sensor to regulate mitochondrial fission and mitophagy (Toyama et al., 2016). Ribeiro et al. further demonstrated that AMPKα1 regulated mitochondrial fusion and fission markers, up-regulated Mfn2 and downregulated Drp1, suggesting that AMPKα1 negatively regulated osteogenesis and alleviated pathological bone loss (Ribeiro et al., 2023). Nishikawa et al. found that Opa1 deficient osteoclast precursor cells failed to undergo efficient osteoclast differentiation and exhibited abnormal ridge morphology, suggesting that Opa1 affected osteoclast differentiation by regulating mitochondrial fusion (Nishikawa et al., 2022).

### 2.7 Mitochondrial transfer

In recent years, extracellular vesicles (EVs) have gained widespread attention as a key carrier for intercellular communication. EVs are able to encapsulate different cargoes from parental cells including lipids, proteins and even genetic material (e.g. mRNA, miRNA) into the receptor cells (van Niel et al., 2022). Interestingly, recent studies have found that mitochondria are also able to wrap into EVs for intercellular mitochondrial transfer. For example, macrophage-derived EVs transferred mitochondria to adipocytes, facilitating the adipocyte-myofibroblast transition in epidural fibrosis (Hua et al., 2024). Another study has shown that cardiac fibroblasts (CFs) participate in the inflammatory response after myocardial infarction by transferring damaged mitochondrial components via small EVs (sEVs), which promote macrophage inflammatory activation (Zhao et al., 2025). In addition, macrophages in periodontitis have also been shown to be able to regulate target cell function through mitochondrial transfer, thereby participating in periodontitis progression.

Yan et al. discovered *in vivo* and *in vitro* experiments that mitochondria are transferred from macrophages to bone marrow mesenchymal stem cells (BMSCs) via mitochondria-rich EVs (MEVs). This inhibits osteogenesis in BMSCs and exacerbates bone loss in periodontitis. The researchers constructed an inflammatory macrophage model of periodontitis and found extensive mitochondrial damage in macrophages, characterized by fragmented morphology, lower membrane potential and more ROS. The presence of MEV was demonstrated by mitochondrial labelling and the entry of these damaged mitochondria into BMSCs disrupted their mitochondrial dynamics as evidenced by the up-regulation of lipid carrier protein 2 (LCN2), OMA1 degradation, and accumulation of OPA1. Abnormalities of mitochondrial dynamics in BMSCs were accompanied by morphology changes, which ultimately led to impaired osteogenesis in BMSCs (Yan et al., 2025). This study elucidated the mechanism by which macrophages promote alveolar bone resorption in periodontitis patients through the transport of mitochondrial vesicles, providing a new perspective for inhibiting alveolar bone resorption in periodontitis. In addition, mesenchymal stem cells (MSCs) are able to influence macrophage function through releasing mitochondria. Phinney et al. found that MSCs release arrestin domain-containing protein 1-mediated MVs (ARMMs), thus unloading mitochondria to transfer mitochondria to macrophages. On the one hand, MSCs can effectively remove depolarized mitochondria and prevent apoptosis. On the other hand, macrophages phagocytose the mitochondria obtained from MSCs and activate mitochondrial fusion proteins (e.g., MFN1, MFN2, and OPA1) to perform mitochondrial fusion, which restores the mitochondrial membrane potential and can increase the energy supply to their macrophages (Phinney et al., 2015). As for the mechanism that causes mitochondrial translocation, it has been suggested that oxidative stress is one of the stimuli that releases mitochondria. Prolonged exposure to 21% O2 significantly increased mtROS levels and increased Parkin and Pink1 kinase expression. Activated Pink1 targets the degradation of Miro, which allowed the release of mitochondria from the cytoskeleton (Jackson et al., 2016).

Based on the fact that altered M1 polarization of macrophages accompanied by altered mitochondrial function is an important cause of progressive periodontitis, Liu et al. explored the potential of apoptotic cell-derived EVs (ApoEVs) to modulate the mitochondrial function of macrophages. Periodontal ligament-derived mesenchymal stem cells (PDLSCs) are a class of mesenchymal stem cells derived from the periodontal ligament (PDL), which are considered to be the main functional stem cells responsible for regeneration of alveolar bone, periodontal ligament and dentin (Lei et al., 2022). The results showed that ApoEVs derived from PDLSCs were able to be phagocytosed by macrophages and that the mitochondrial component of them enhanced the mtDNA levels and ATP production capacity of macrophages damaged in inflammatory environment (Liu et al., 2024c). ApoEVs offer the possibility to ameliorate macrophage mitochondrial damage in periodontitis and indirectly promote periodontal tissue regeneration by modifying macrophage phenotype.

Migrasomes are miniature EVs formed at the posterior end of migrating cells. Similar to other EVs, they recruit cellular contents including mitochondria and are enriched for many biomolecules such as growth factors, cytokines, chemokines and morphogens (Jiao et al., 2021). Specifically, the release of migrants is dependent on cell migration and the TSPAN4 protein, which is involved in target cell migration-related functions or signaling. Damaged mitochondria can be translocated to migrasomes and subsequently discarded from migrating cells under mild mitochondrial stress, a process named mitocytosis (Zhang et al., 2022a). Mechanistically, mtROS are produced in response to mitochondrial stressor induction. Damaged mitochondria move towards the pericellular perimeter mediated by KIF5B, while myo-19 tethers the mitochondria to cortical actin, which is tightly associated with the plasma membrane. During this process, mitochondria undergo Drp1-mediated division (Jiao et al., 2021). Osteoclast maturation is dependent on the migratory fusion of pro-osteoclasts to form multinucleated cells, and Lampiasi et al. found that RANKL-stimulated osteoclast differentiation of RAW 264.7 cells induced the formation of vesicles similar to’migrasomes’ (Lampiasi et al., 2021). Macrophages and neutrophils are known to produce migrasomes as a means of mitochondrial quality control (Jiang et al., 2023c), but the biological role of migratory bodies for macrophages in periodontitis needs to be further investigated.

Based on the phenomenon that cells can take up mitochondria from their environment or from other cell types, mitochondrial transplantation was invented as an emerging bionanotechnology. It refers to the reversal of diseases associated with mitochondrial disorders by transferring healthy mitochondria into damaged cells to restore cellular function (Liu et al., 2022b). A patent (CN113633662) has been issued for the mitochondrial transplantation of PDLSCs for the treatment of periodontitis, using a kit and differential centrifugation to extract mitochondria from healthy human PDLSCs. Healthy mitochondria captured by inflammation-injured human PDLSCs were able to enhance the osteogenic differentiation of human PDLSCs. In addition, macrophage differentiation towards the M2 type was promoted through the immunomodulatory function of PDLSCs. In addition, the patent was also demonstrated to attenuate inflammatory periodontal tissue injury and alleviate periodontitis bone loss in mouse experiments.

In summary, the transfer of damaged mitochondria from macrophages to target cells in the periodontitis setting may accelerate periodontitis destruction. However, acceptance of healthy mitochondria can improve the energy supply of macrophages and promote their polarization towards M2, providing a new strategy for the treatment of periodontitis. In addition, migrasomes, as an emerging EV type, may also be involved in osteoclast formation, and their role in periodontitis needs to be further investigated.

## 3 Targeting mitochondria in macrophages for the treatment of periodontitis

Traditional periodontitis treatment modalities, like supragingival scaling, SRP, antibiotics, surgical interventions have limitations such as high recurrence and incomplete plaque removal (Jiang et al., 2023b). Recently, research has increasingly proved that mitochondria play a role in macrophage function changes during periodontitis, leading to mitochondrial - targeted therapy emerging as a new treatment strategy. Common approaches involve antioxidants, regulating mitophagy, and improving metabolic reprogramming. Additionally, novel methods such as photodynamic therapy (PDT) and nanomaterials are being innovatively applied in macrophage - targeted mitochondrial therapy.

### 3.1 Antioxidants

Oxidative stress is crucial in the progression of periodontitis. Antioxidants are therapeutic in periodontitis by attenuating tissue destruction, reducing inflammation and promoting regeneration. More importantly, antioxidants may act as mediators to alleviate the systemic oxidative stress and inflammatory state induced by periodontitis-associated systemic diseases (Deng et al., 2024).

Resveratrol (trans-3,4′,5-trihydroxystilbene) is a common natural polyphenolic compound found in a variety of plants, including grape skins, blueberries, and raspberries, and has been widely demonstrated to have free radical scavenging properties (Huo et al., 2022). *Porphyromonas gingivalis* infection has been shown to decrease the clearance of damaged mitochondria from macrophages, disrupt the mitochondrial quality control system, and promote the secretion of inflammatory cytokines (Montava-Garriga and Ganley, 2020). Further, Jiang demonstrated that the scavenging effect of resveratrol on mtROS may be due to the up-regulation of PINK1 to cause mitophagy, thus ensuring mitochondrial quality (Jiang et al., 2023a), which may suggest its inhibitory effect on periodontitis with *Porphyromonas gingivalis* as the main causative agent.

In addition, it has been shown that the antioxidant effect of resveratrol is related to its ability to activate NRF2 to induce the expression of various antioxidant genes, such as hemoglobin oxygenase-1 and NAD(P)H (Soufi et al., 2012; Alavi et al., 2021). Ikeda et al. applied monomeric resveratrol and its dimeric form to two distinct groups of murine periodontitis models, respectively and found that the administration of resveratrol to both groups could reduce the periodontal bone loss, but the effect was stronger in the dimeric group. In addition, the dimer group showed a decrease in the level of the pro-inflammatory cytokine IL-1β (Ikeda et al., 2022). The promotional effect of resveratrol on bone healing in periodontitis may be related to the fact that Nrf2 regulates the cellular redox state by controlling the expression of oxidation-responsive genes, thereby inhibiting RANKL-induced OC differentiation. Similarly, curcumin and radicicicol have been shown to inhibit periodontitis bone loss by activating Nrf2 in part by inhibiting macrophage osteoclast differentiation (Hyeon et al., 2013).

The NLRP3 inflammasome complex regulates IL-1β release and resveratrol inhibits NLRP3 inflammasome activation by decreasing mtROS production and mtDNA release, which in turn decreases macrophage proinflammatory cytokine release (Chang et al., 2015). Similarly, Adhikari et al. observed that resveratrol treatment reduces pro-inflammatory cytokines and increases alveolar bone volume in extraction sockets compared to the control group (Adhikari et al., 2021). Furthermore, Shi et al. demonstrated that resveratrol could transform inflammatory macrophages from the M1 to the M2 phenotype by activating p-STAT3 and down-regulating the expression of p-STAT1, thereby reconstructing the immune microenvironment of periodontitis (Shi et al., 2021a).

Lignocaine (LUT) is a natural flavonoid derived from vegetables, fruits and herbs with potent anti-inflammatory activity and the ability to induce macrophage polarization towards M2 *in vitro* experiments (Wang et al., 2020b). A growing number of studies have shown that LUT can attenuate alveolar bone loss and reduce inflammatory factor release in periodontitis (Casili et al., 2020; Arampatzis et al., 2023). To further elucidate the mechanism of LUT action, Ma et al. experimentally showed that LUT may regulate mitochondrial dynamics and promote macrophage M2 polarization by targeting JAK2/STAT3 signaling. Specifically, LUT down-regulated Drp1 to reduce mitochondrial division by inhibiting the JAK2/STAT3 pathway and enhanced mitochondrial fusion through up-regulation of Mfn, which synergistically improved mitochondrial function and further supported macrophage M2 polarization (Ma et al., 2025).

Hydroxytyrosol (HT), a natural phenolic compound with antioxidant capacity, can inhibit mitochondrial dysfunction by decreasing the cleavage of OPA1 and elevating the phosphorylation of AKT and GSK3β (Cai et al., 2019). RANKL plays an active role in osteoclastogenesis through the activation of multiple signaling events including the MAPK pathway. The MAPK signaling pathway is a potential target for periodontal therapy, and ERK and JNK are downstream targets of this signaling pathway (Hu et al., 2024). Zhang et al. demonstrated that HT treatment attenuated macrophage mtROS and MMP levels, and attenuated mitochondrial oxidative stress injury. In addition, HT was able to inhibit osteoclast differentiation by suppressing the ERK/JNK pathway in mitochondria, thereby ameliorating bone loss in a murine periodontitis model (Zhang et al., 2021). Similarly, Jiang et al. synthesized 3-methyl-1H-indol-1-yl dimethylcarbamodithioate (3o), which could attenuate periodontitis by inhibiting inflammatory responses and suppressing osteoclast differentiation. On the one hand, 3o significantly reduced osteoclast differentiation by inhibiting the MAPK signaling pathway. On the other hand, 3o was able to restore mitochondrial function, as evidenced by a decrease in the levels of mtROS, MMP and ATP. And the improvement of inflammatory response and bone resorption after 3o treatment was also found in a murine periodontitis model (Jiang et al., 2024).

CoQ10, an endogenous small-molecule organic compound with potent antioxidant capacity, is an essential component of the mitochondrial ETC (Brown and McCarthy, 2023). CoQ10, as an important fat-soluble antioxidant, protects mitochondrial membranes, proteins, and DNA from ROS damage (Hargreaves et al., 2020). It has been demonstrated that CoQ10 can inhibit the activation of NLRP3/IL1β signaling by suppressing excess ROS production, thereby reducing macrophage pro-inflammatory cytokine secretion and M1 polarization (Pan et al., 2024). Moon et al. found that CoQ10 significantly inhibited the genetic markers of osteoclast differentiation, such as NFATc1, TRAP and the gene expression of osteoclast-associated immunoglobulin-like receptors. In addition, CoQ10 significantly inhibited the H2O2-induced IκBα, p38 signaling pathway and suppressed osteoclastogenesis (Moon et al., 2013). Reduced levels of CoQ10 were found in gingival biopsies of approximately 80% of patients with periodontitis (Soni et al., 2012). CoQ10 has been proposed by some investigators as an adjunctive treatment to SRP, with desirable results in clinical trials (Sharma et al., 2016). Clinical trials have shown that oral administration of CoQ10 to patients with periodontal disease increased the concentration of CoQ10 in diseased gingiva and was effective in suppressing advanced periodontitis (Prakash et al., 2010).

Based on the role of the NLRP3 inflammasome, researchers developed a new strategy for the treatment of periodontitis. Artesunate (ART) is a peroxide from the traditional plant Artemisia annua, which is effective in preventing malaria and treating tuberculosis. Recent studies have demonstrated that ART and its components have anti-inflammatory and immunomodulatory effects on various skeletal diseases, but the exact mechanism is still unclear. Wang et al. found that ART was able to inhibit osteoclastogenesis by suppressing NLRP3 inflammasome activation and enhance osteogenic potential by attenuating the expression of inflammatory factors under inflammatory conditions. In addition, ART enhanced osteogenic potential by attenuating the expression of inflammatory factors. These results confirming the possible therapeutic role of ART in periodontitis (Wang et al., 2023b).

### 3.2 Regulation of mitophagy

The role of mitophagy and division in the pathogenesis of periodontitis is gaining increasing attention. PINK1 has been shown to protect cells from oxidative stress by promoting the degradation of damaged mitochondria through mitophagy. In periodontitis, PINK1 deficiency is thought to contribute to the induction of osteoclast overproduction (Jang et al., 2024). Spermidine (SPD), an endogenous polyamine, is thought to modulate the PINK1/Parkin pathway and restore mitophagy (Zimmermann et al., 2024). Reversing the excessive osteoclast production induced by PINK1 deficiency provides a new therapeutic strategy for severe periodontitis accompanied by fulminant osteolysis.

Mitochondria are the primary site of iron utilization, and iron deficiency leads to impaired heme synthesis and iron-sulfur cluster assembly, further affecting the assembly and function of the mitochondrial respiratory chain. Studies have demonstrated that osteoclasts require high iron uptake to meet their high energy demands. Transferrin receptor 1 (Tfr1) mediates cellular iron uptake. Osteoclasts in Tfrc knockout mice have suppressed expression of mitochondrial complexes I and II, and reduced mitochondrial membrane potential, which is manifested as a decrease in osteoclast function (Das et al., 2022). Based on the critical role of iron in osteoclast function, the use of iron chelators to reduce osteoclast differentiation has become a therapeutic direction for periodontitis. Zhu et al. demonstrated that desferrioxamine reduced the progression of periodontitis, osteoclastogenesis, and alveolar bone loss in a murine periodontitis model (Zhu et al., 2024). Previous studies have shown that high iron uptake promotes PGC-1β-regulated mitochondrial biogenesis and provides energy for osteoclast function. Based on this, Li et al. applied Isobavachin to attenuate osteoclastogenesis and periodontitis-induced bone loss by promoting Fpn1-mediated iron efflux from osteoclasts and inhibiting PGC-1β-mediated mitochondrial biogenesis in osteoclasts (Li et al., 2024b).

### 3.3 Regulation of metabolic reprogramming

A shift in macrophage metabolism from OXPHOS to glycolysis has been demonstrated in the setting of periodontitis (Fleetwood et al., 2017). *In vitro* and vivo experiments, the glycolysis inhibitor 2-Deoxy-d-glucoses significantly inhibited LPS-induced macrophage apoptosis, thereby attenuating the inflammatory response and bone resorption in periodontal lesions. Further, He et al. demonstrated that the regulatory effect of glycolysis on macrophage apoptosis may be mediated through the AMPK/SIRT1/NF-κB signaling pathway, which provides a potential new target for PD therapy (He et al., 2023b).

Disruption of the TCA cycle and up-regulation of the glycolysis and pentose phosphate pathways are characteristic of M1 macrophages. Apabetalone is a selective bromodomain and extra-terminal (BET) protein inhibitor targeting epigenetic regulators of bromodomain 2 and gene expression (Ray et al., 2020). Recent studies have demonstrated that Apabetalone inhibited glycolysis by suppressing the transcription and protein expression of hexokinase 2, glucose transporter 1 and phosphofructokinase-2/fructose-2,6- bisphosphatase 3 (PFKFB3) in a dose-dependent manner. In addition, Apabetalone was able to complement the disruption of the TCA cycle, thereby inhibiting macrophage M1 polarization and attenuating periodontitis bone resorption (Bian et al., 2025).

Based on the effect of macrophage mitochondrial metabolism on macrophage polarization, He et al. developed a molybdenum-containing bioactive glass-ceramic (Mo-BGC) scaffold with functions such as continuous release of Mo ions. The results indicated that the Mo-BGC scaffold could regulate glycolysis and the production of circulating metabolites of TCA, which would promote M2 macrophage polarization and periodontal tissue regeneration (He et al., 2022).

### 3.4 Emerging therapeutic approaches

#### 3.4.1 Nanomaterials

Nanomaterials show great potential in the field of periodontitis treatment. In the experiment of Hunagfu et al., resveratrol (RES) and 20(S)-protopanaxadiol (PPD) were successfully self-assembled into RES@PPD nanoparticles (NPs) by the phenolic resin reaction. The novel NPs have the advantage of nanoscale size and easy penetration deep into periodontal pockets. Meanwhile, the combined application of PPD and RES enhanced the anti-inflammatory and antioxidant properties of the nanocomposites, scavenging ROS to promote macrophage M2 polarization; RES@PPD nanoparticles significantly reduced the levels of pro-inflammatory cytokines in a murine periodontitis model, and were able to inhibit inflammation in periodontal tissues effectively (Huangfu et al., 2023).

The development of periodontitis and the activation of M1 macrophages are dependent on the sustained over-opening of mPTP, which is mainly caused by mitochondrial Ca2+ overload (Qi et al., 2021). He et al. first synthesized PAMAM decorated with PEGylated TPP (PAMAM-PEG-TPP)., which loaded with BAPTA-AM to make Mitochondrial Calcium Ion Nanogluttons. Triphenylphosphine (TPP), a lipophilic cation, is a widely used mitochondrial targeting molecule that can accumulate in mitochondria driven by mitochondrial membrane potential (Li et al., 2022a). BAPTA-AM is a calcium ion-selective chelator, which avoids calcium overload-induced mPTP opening and M1 macrophage activation. The results showed that after treatment with these Mitochondrial Calcium Ion Nanogluttons, localized periodontitis in mice was alleviated and accompanied by a reduction in osteoclast activity and bone loss (He et al., 2023a).

Li et al. synthesized controlled drug release nanoparticles (MitoQ@PssL NPs) by encapsulating the autophagy enhancer mitoquinolone (MitoQ) into amphiphilic polymer nanoparticles (PssL NPs). ROS-sensitive dynamic covalent bonds were formed in response to the specific high ROS microenvironment of periodontitis. The autophagy enhancer MitoQ could achieve responsive release in the periodontal inflammatory microenvironment and effectively induce mitophagy via the PINK1-Parkin pathway (Li et al., 2022b).

Yan et al. Cerium-doped zeolite imidazole framework-8 (Ce@ZIF-8) nanoparticles (NPs) could alleviate periodontitis by affecting metabolic reprogramming. It was shown that Ce@ZIF-8 NPs inhibited HIF-1α, suppressed the glycolytic process and promoted M2 polarization in macrophages (Yan et al., 2023).

Shaheen et al. designed a micellar nanocarrier of CoQ10. It demonstrated superior antioxidant activity in combination with conventional treatment of periodontitis with SRP over single mechanical methods and is expected to be used in the treatment of moderate periodontitis (Shaheen et al., 2020).

#### 3.4.2 Photodynamic therapy

In addition to pharmacological treatments targeting mitochondria, PDT has also been explored for ameliorating oxidative stress in periodontitis. Jiang et al. conducted *in vivo* and *in vitro* experiments and found that methylene blue-mediated PDT was able to induce macrophage apoptosis and reduce bone loss in periodontitis rats by modulating ROS levels and decreasing mitochondria-dependent apoptosis (Jiang et al., 2019). Lopez et al. conducted a clinical trial in which 44 patients with periodontitis were sterilized with PDT using a 635 nm wavelength laser source and a light-activating substance (methylene blue), and significant reductions in parameters such as the plaque index (PI), bleeding on probing (BOP), and probing depth (PD) were seen at 3 months (12 weeks) after treatment (Lopez et al., 2020). This further demonstrates PDT as an effective adjunctive treatment for periodontitis.

Antimicrobial photodynamic therapy (aPDT) is an alternative treatment in which lasers and different photosensitizers are used to eradicate periodontal pathogenic bacteria in periodontitis (Bourbour et al., 2024). Many studies have shown that SRP plus aPDT has more significant results in probing depth improvements compared to SRP alone (Chambrone et al., 2018).

## 4 Conclusions and future perspective

In this review, we focus on the mechanism of mitochondrial dysfunction in periodontitis on macrophages and the application of targeting mitochondria in periodontitis treatment. In terms of the pathogenesis of periodontitis, dental plaque and the host immune response have been the focus of research and mitochondria have been shown to be involved in the inflammatory response in recent years. Mitochondrial dysfunction, including oxidative stress injury, reduced biosynthesis, dynamic injury, abnormal mitophagy and even mitochondria-mediated alterations in cellular metabolic patterns, is involved in macrophage function and the development of periodontitis, further enriching the understanding of the pathogenesis of periodontitis.

With more and more studies, the understanding of mitochondrial dysfunction in macrophages in the setting of periodontitis is progressively deepening to the molecular level. Oxidative stress with overproduction of mtROS, open mtDNA leakage of mPTP, down-regulation of PINK1/Parkin-mediated mitophagy in periodontitis environment, activation of NLRP3 inflammasome caused by mtDNA and aberrant expression levels of Drp1, Fis1, Mfn1 and Mfn2 related to mitochondrial fusion and division are identified in researches. These evidences provide a basis for mitochondria-targeted therapy, which provides a possible adjunct to periodontitis treatment. Meanwhile, the combination of mitochondria-targeted therapy and nanomaterials enables the drugs to penetrate deep into the periodontal pockets to achieve precise drug delivery and enhance drug delivery efficiency. In addition, mitochondrial transfer has been found in periodontitis, but its specific mechanism and role remain to be studied.

However, the current investigation about the role and application value of mitochondria in periodontitis still has some limitations. Most of the current studies on mitochondria focus on cellular experiments, and part of studies on the correlation between mitochondria and diseases are mostly applied in the fields of cancer, cardiovascular diseases and neurological diseases, while relatively few studies have been conducted in periodontitis. The complex mechanisms of mitochondrial dysfunction in the etiology and progression of periodontitis remain to be further elucidated. In addition, most of the mentioned mitochondria-specific therapies above are still in the preclinical stage, and their long-term effects and adverse reactions need to be further investigated.

In conclusion, there is a broad link between mitochondrial dysfunction and macrophage function in periodontitis, and mitochondrial dysfunction contributes to periodontitis progression and bone destruction. Future research should focus on the mitochondrial heterogeneity of different cell types in the periodontitis microenvironment, and the clinical translation of targeted mitochondrial therapies for periodontitis is a promising area.
